# Association study between vitamin D receptor gene polymorphisms and asthma in the chinese han population: a case-control study

**DOI:** 10.1186/1471-2350-10-71

**Published:** 2009-07-21

**Authors:** Ahlem Saadi, Guimin Gao, Huaichen Li, Chunhua Wei, Yaoqin Gong, Qiji Liu

**Affiliations:** 1 Key Laboratory for Experimental Teratology of the Ministry of Education and Department of Medical Genetics, Shandong University School of Medicine, Jinan, Shandong 250012, PR China; 2 Department of Respiratory Internal Medicine, Shandong Province Hospital, Jinan, Shandong 250001, PR China; 3 Weifang Asthma Hospital, Weifang, Shandong 264100, PR China

## Abstract

**Background:**

Modulation of the immune system is one of the principal roles of Vitamin D, for which the effects are exerted via the vitamin D receptor (VDR). Importantly, variants in the VDR gene have been susceptible in the past to raise the risk of asthma in several populations. These effects of VDR allelic markers remain speculative in the Chinese Han population.

**Results:**

A case-control study of 1090 individuals including 567 asthmatic patients was realized on five SNPs within the VDR gene. Only rs7975232 (ApaI) marker showed a significant association with asthma (P = 0.009). Haplotype analysis of the five VDR polymorphisms showed a significant association with asthma (global-p value = 0.012).

**Conclusion:**

Although the susceptibility of VDR gene variants with asthma could not be confirmed for all SNPs tested in this study, the significant association obtained for rs7975232 provides evidence for a previously unknown report about the Chinese Han population and may raise the susceptibility of VDR to be a candidate gene for asthma.

## Background

Recent discoveries made in the field of molecular biology have reported that vitamin D derived from diet and supplements is not a vitamin, but a steroid with immunosuppressive properties when elevated [1]. It has also been linked with lessening symptoms of autoimmune diseases like multiple sclerosis [2], Crohn's disease [3], and rheumatoid arthritis [4].

However, the effects of this steroid-like molecule are essentially exerted via its cognate the vitamin D receptor (VDR). In addition, molecular modeling findings have defined an interaction between the hormonal 1, 25-dihydroxyvitamin D3 form and its receptor through a ligand-binding domain (LBD) [5]. The activated VDR plays then a fundamental role in the body by regulating numerous primary target genes. Three years before, a Canadian study has identified on a large scale that VDR may transcribe more than 900 genes [6]. In fact, the interaction of 1, 25(OH)_2_D_3 _with VDR and its cofactors modulates many biological activities of the neural, immune, and endocrine systems; including calcium and phosphorous homeostasis, apoptosis and cell differentiation. For instance, a previous study has demonstrated that VDR rs7975232 polymorphism is associated with hip bone mineral density (BMD) in a group of 260 healthy postmenopausal Chinese Han women [7].

Besides to this classical action, and due to the pleiotropic effect the 1, 25(OH)_2_D_3_-VDR complex exerts, its genetic variants have been found to be associated with a variety of diseases/phenotypes, in which the risk can often depend on inter-individual variability or genetic differences within VDR protein; such like the heterogeneous phenotype of asthma. In fact, since VDR is located within the region q13-26 of chromosome 12 previously linked to asthma or related phenotypes in different populations [8-12], it is considered to be one of the candidate genes of asthma. A number of association studies were previously conducted in different populations and ethnic groups. Two of them have suggested associations among VDR polymorphisms and asthma. The first one was a family-based study on a founder French population from northeastern Quebec, accompanied by a second Canadian study, using both the childhood asthma management network as family-based study and the Healthy women from European ancestry as a case-control study [13,14]. Another research among a German population has also tested the same hypothesis, however, there was no preferential transmission of VDR variants to children with asthma [15]. Assays were also done on animal models, where an experimental allergic asthma was induced in VDR knockout and wild-type (WT) mice. As expected, WT mice developed symptoms of airway inflammation with an influx of eosinophils, elevated Th2 cytokine levels, mucous production, and airway hyperresponsiveness [16].

Thus, we sought to replicate the association previously described between the VDR gene and asthma among a Chinese Han population. Five Single nucleotide polymorphisms (SNPs) were selected, genotyped and statistically analyzed in a case-control study including 1090 Chinese individuals. Our results demonstrated that VDR rs7975232 (ApaI) variant was associated with asthma susceptibility in the cohort studied.

## Methods

### Subjects

All subjects were Chinese of Han ethnic origin living in the same urban area. This group form 99% of the local population of Shandong, an eastern coastal province of China. Of note, the ethnicity of the group was also evident from the similar lifestyle and dietary habits. This research is one of the association studies carried out in our Institute, where several asthma susceptibility genes with different locations and functions were being tested to identify some novel linkages to the complex phenotype of asthma in the Chinese Han population. For that purpose, the process of collecting the samples was done thanks to collaboration with a number of provincial Hospitals. Our primary number of samples has increased since three years [17-19] to a larger number, of which 567 cases and 523 controls were collected for this study. Detailed information about age, sex, clinical characteristics and original medical institution were obtained for all participants (see Table 1). Healthy subjects, matched for age, were sampled from both Shandong Provincial hospital and Qilu hospital in Jinan city, where they had undergone pulmonary function tests, by which no doctor-diagnosed asthma nor history of asthma or other pulmonary diseases were noted. Whereas, affected subjects were recruited from both Qilu Hospital and Weifang Asthma Hospital. The 314 females and 253 males all confirmed asthma diagnosis by spirometry, which was performed according to American Thoracic Society recommendations (ATS, 1987); while no other atopy-related diseases or allergy phenotypes were available for those patients. Blood samples were obtained from all participants with informed consent. Genomic DNA was extracted from peripheral blood leukocytes by a standard salting-out method. The study was approved by the ethics review committee for human studies at School of Medicine, Shandong University.

**Table 1:** Baseline characteristics of the asthmatic patients with asthma and controls.

**Characteristics**	**Patients(n = 567)**	**Controls (n = 523)**
**Samples origins and**	Weifang **(n = 424) **195/229	SPH **(n = 308) **217/91
**Sex distribution(male/female)**	Qilu Hospital**(n = 143) **58/85	Qilu Hospital **(n = 215) **143/72
**Age: mean (range)**	41.36 (4–77)	44.18 (18–79)
**FEV1, % predicted**	56.48 ± 16.8	85.98 ± 12.2
**Changes in FEV1 by bronchodilator, %**	28.28 ± 14.6	4.38 ± 3.9
**Total frequency of females**	55.4%	33.3%
**Total frequency of males**	44.6%	66.6%

SPH stands for Shandong Provincial Hospital

### SNPs Selection and Genotyping

In this Case-control study, we tested five polymorphisms of VDR gene, which maps to chromosome 12q13.11, consists of 9 exons with at least 6 isoforms of exon 1, spans 63.5 kb, and encodes a 427-amino acid protein [20]. All variants are candidate SNPs, which are known to have a specified extent for asthma and/or other pulmonary diseases described in the earlier researches among different populations: rs2228570 or FokI [21], rs3782905 [13,14], rs1544410 or BsmI [14], rs7975232 or ApaI [14,22], and rs731236 or TaqI [14,15,23]. Two synonymous sites were primary selected, rs2228570 (a T/C transition polymorphism in exon2) and rs731236 (a single base change C to T in codon 352 at the 3' end of the VDR gene). Further, the three other sites were investigated in the non-coding regions of VDR gene, including rs7975232 and rs1544410 both in intron 8, and rs3782905 in intron 2. All markers were analyzed by a standard PCR-restriction fragment length polymorphism (PCR-RFLP) method. The different loci were recognized by the following restriction endonucleases (New England Biolabs NEB-China, Beijing): FokI for C/T at 37°C in exon 2, TaqI for T/C at 65°C in exon 9, BsmI for G/A at 65°C and ApaI for C/A at 37°C in intron 8, respectively and DdeI for C/G at 37°C in intron 2 (primers, product lengths and cycling conditions are all summarized in Table 2).

**Table 2:** Different loci selected within the vitamin D receptor sequence and their corresponding primers used during PCR-RFLP method.

Location	Locus	Alleles	PCR primer	PCR Product (bp)	Restriction enzyme	RFLP products(bp)
Exon 2	rs2228570	C/T	F: 5'-CCTGGCACTGACTCTGGCTCTG-3' R: 5'-GGCTCCCTTCATGGA AACACCT-3'	270	FokI	207/63
Intron 2	rs3782905	C/G	F: 5'-AAGACATGGTGTCTGCTTCA-3' R: 5'-GGTTAGATCGATATGTTTGA-3'	304	DdeI	223/81
Intron 8	rs1544410	G/A	F: 5'-GGTGGGACTGAAGAAGCTGAAC-3' R: 5'-CTTTGGACCTCATCACCGACAT-3'	613	BsmI	357/256
Intron 8	rs7975232	A/C	F: 5'-GTAGAATAGAAGGAGGGAAGC-3' R:5'-AGAGGCAGCGGTACTGCTTGGAGTG-3'	662	ApaI	424/238
Exon 9	rs731236	T/C	F: 5'-GTAGAATAGAAGGAGGGAAGC-3' R: 5'-AGCTTCATGCTGCACTCAGGCT-3'	690	Taq^α^I	504/186

### Statistical Analysis

Single nucleotide polymorphisms were assessed for both genotypic and allelic association analysis among asthma patients and healthy controls. The genotype data of all tested SNPs were then used to estimate Hardy-Weinberg equilibrium by comparison of genotype frequencies within the two groups by a χ^2^-test. P-values were calculated with SPSS statistical software (SPSS Statistics 17.0; 2008 SPSS Inc, Chicago, IL), where Fisher's exact test was applied to analyze the comparison of the frequencies of discrete variables between cases and controls. The odds ratios (ORs) with 95% confidence intervals (95%CI) were also calculated during the single site analysis to estimate risk of asthma associated with the VDR polymorphic genotypes. An adjustment for multiple tests by Bonferroni correction in which the P-values are multiplied by the number of comparisons was later used to control the false discovery rate. Haplotypes and their frequencies were also constructed using the online software SHEsis [24], in which differences of haplotype distribution between patients and controls is assessed by the Monte Carlo method. The differences in pairwise distribution between respective pairs of SNPs tested were assessed by the statistical measure of LD based on calculating D' using Haploview 4.1[25]. Further, a graphical representation of different genotype data obtained with SNP rs7975232 was produced, using a powerful program for analyzing graphs (Graphical Analysis 3.4), in which the genotype frequencies of cases and controls obtained with this site were diagrammatically compared.

## Results

For the five polymorphisms tested in this study within the VDR gene sequence, genotype distributions were in Hardy-Weinberg equilibrium in both patients and controls (all P-value > 0.05). The first association result with the coding polymorphisms VDR/rs2228570 and VDR/rs731236, however negative, was important since it excluded the susceptible risk. In fact, the statistical analysis of these two SNPs did not call for any association with asthma (P > 0.05). In the second part, three other SNPs were tested within non-coding regions of VDR sequence, VDR/rs3782905, VDR/rs7975232 and VDR/rs1544410. In contrast to the marker rs7975232, the role of the intronic variants rs3782905 and rs1544410 in asthma could not be confirmed. Hence, among the five markers studied, only intronic SNP rs7975232 (ApaI) was standing out as a significant polymorphism with an increased risk in one group compared to the other (The genotype analysis and the results of association tests for the five markers are all represented in Table 3).

**Table 3:** Genotypic and allelic association analysis of vitamin D receptor single-nucleotide polymorphisms in the Chinese asthma study.

**SNPs ID**	**Controls (n = 523)**	**Asthmatics (n = 567)**	**OR (95% CI)**	***P *value**	**Genotype**	**Controls (n = 523)**	**Asthmatics (n = 567)**	***P *value**
rs2228570					CC	152 (0.29)	193 (0.34)	
C	569 (0.54)	644 (0.57)	1.00		CT	265 (0.51)	258 (0.46)	
T	477 (0.46)	490 (0.43)	1.10 (0.93–1.30)	0.261	TT	106 (0.20)	116 (0.20)	0.161
rs3782905					CC	378 (0.72)	415 (0.73)	
C	895 (0.85)	972 (0.86)	1.00		CG	139 (0.27)	142 (0.25)	
G	132 (0.15)	162 (0.14)	0.88 (0.69–1.13)	0.920	GG	6 (0.01)	10 (0.02)	0.754
rs1544410					GG	472 (0.90)	515 (0.91)	
G	995 (0.95)	1079 (0.95)	1.00		GA	51 (0.01)	49 (0.08)	
A	51 (0.05)	55 (0.05)	1.00 (0.68–1.48)	0.977	AA	0 (0.00)	3 (0.01)	0.208
rs7975232					CC	247 (0.47)	310 (0.55)	
C	710 (0.68)	837 (0.74)	1.00		CA	216 (0.41)	217 (0.38)	
A	336 (0.32)	297 (0.26)	1.33 (1.10–1.60)	**0.002**	AA	60 (0.12)	40 (0.07)	**0.009**
rs731236					TT	471 (0.90)	510 (0.90)	
T	993(0.95)	1073 (0.95)	1.00		TC	51 (0.09)	53 (0.09)	
C	53(0.05)	61(0.05)	0.94 (0.64–1.37)	0.446	CC	1 (0.01)	4 (0.01)	0.743

The *P *values < 0.05 are indicated in bold.

Allelic and genotypic frequencies of both controls and asthmatics are shown in parentheses.

**Figure 1:**
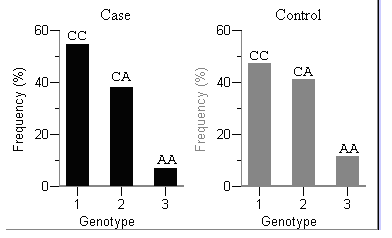
**Frequency distribution of VDR-rs7975232 within the two groups (case/control) in the Chinese population studied**. The bars represent the frequencies of the 3 genotypes, CC, CA, AA, which are labeled by 1, 2, 3, respectively, in each group. We can clearly see that AA genotype is underrepresented in the group of asthmatic patients.

Importantly, rs7975232 has shown a strong association with asthma in our Chinese samples, and the risk conferred by it was slightly excessive (P = 0.009; Odds Ratio = 1.33; 95% CI: 1.10–1.60). In addition, after graphical representation of its different genotypic frequencies distribution, the minor allele has demonstrated protective effect in a recessive model in affected subjects, of which the corresponding homozygote genotype AA was underrepresented in the case data comparing to the control data (Figure. 1).

Genotypic and allelic P-values were also calculated for rs7975232 site through a detailed statistical analysis (see Table 4). Also, when the difference for the same variant was adjusted for multiple testing by the Bonferroni method, the global significant association obtained was still persisting (P = 0.045). One other significance level was obtained with AA homozygote individuals (P = 0.004) in this analysis, and a highest odds ratio was also estimated for the same genotype (Odds Ratio = 1.88, 95% CI: 1.22–2.90). Consequently, these results suggest in all likelihood that minor allele of rs7975232 marker is a risk allele for asthma in our Chinese cohort studied.

**Table 4:** Detailed statistical analysis of VDR ApaI site in the Chinese cohort.

	Allelic analysis			Genotypic analysis				
	C	A	CC	CA	AA	χ^2^	Global *P*	P*
Odd	1.00	1.333	1.00	1.249	1.882			
(95%CI)	---	(1.10–1.60)		(0.97–1.61)	(1.22–2.90)			
*P *value	**0.002**		---	0.083	**0.004**	9.367	**0.009**	**0.045**

*P *values < 0.05 are indicated in bold.

P* corresponds to global *P *value adjusted by *Bonferroni *correction for multiple tests.

**Figure 2:**
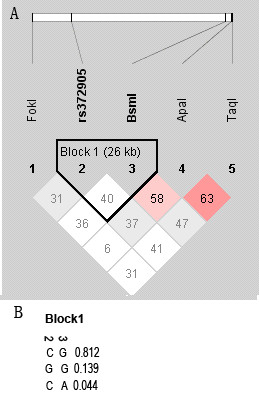
**A. Pairwise linkage disequilibrium map (LD) of VDR markers typed in this case-control study**. The position of each SNP along the chromosome 12 (region q13-11) is indicated on top. The statistical measure of LD or D' × 10^-2 ^is known as the strength of LD between approximately evenly spaced SNPs. Common measure of LD represented here by different numbers on each square and progression of red color. One block was detected among VDR loci studied (Block 1).**B**. Different haplotypes found within the "Block 1" constituted of two SNPs (rs3782905 and rs1544410) and their corresponding frequencies.

Finally, evaluation of LD between each pair of loci has determined one block on the LD plot structure ("Block 1"; see figure 2A/B), which spans a region of 26 kb between two SNPs (rs3782905 and rs1544410); from intron 2 (position 46552434) to intron 8 (position 46526102). However, LD in this region was considered low, which is perhaps due to the low frequency seen with the minor alleles of these two SNPs, since they may not affect the "Block 1". Whereas, further in the 3'-terminal region more significant D' measures were seen between the two pairs rs1544410-rs7975232 and rs7975232-rs731236 (see figure 2A). This can probably explain the association observed with the variant rs7975232 in this region. In addition to this result, to investigate whether rs7975232 or neighboring non-polymorphic loci were causative, haplotype association analysis was later conducted with the complete five-locus haplotype studied (see Table 5). The analysis has shown a significant global association result with asthma (*χ*^2 ^= 14.51, P = 0.012), where the selected haplotype TCGAT has demonstrated the highest significance association (P = 0.0005).

**Table 5:** Five-locus haplotype analysis for transmission disequilibrium test.

Haplotype	Case(freq)	Control(freq)	χ^2^	*P *value
CCGCT	0.316	0.292	1.33	...
CCGAT	0.097	0.108	0.79	...
CGGCT	0.084	0.079	0.13	...
TCGCT	0.282	0.247	3.42	...
TCGAT	0.095	0.142	11.84	0.0005
TGGCT	0.030	0.032	0.09	...
Global			14.51	0.012

Selected haplotype results are shown. Only significant (*P *≤ .05) or global *P *values are listed.

All low frequency haplotypes (<3%) were discarded from the analysis by the software SHEsis.

The order of SNP: rs2228570-rs3782905-rs1544410-rs7975232-rs731236

## Discussion

This research directs an issue to test the hypothesis described in the previous association studies between VDR variants and asthma. Though the susceptibility for asthma disease could not totally be confirmed for all SNPs tested in this study, the significant result obtained with the non-coding polymorphism rs7975232 has reported previously unknown information about the relation existing between VDR gene and asthma in the Chinese Han population. This may also increase the probability of VDR to be a candidate gene for asthma.

Our results represent also a replication of the previous findings in the two U.S. studies, where SNP rs7975232 polymorphism has also shown a significant association with asthma. In the CAMP, seven VDR polymorphisms were genotyped in complete nuclear families from three different ethnic groups included in the analysis. After a stratified genetic analysis by ethnic groups, only intronic variant rs7975232 has shown a significant association with asthma (P = 0.01) [14]. However, the NHS on 517 cases and 519 controls has shown evidence for association with asthma of four out of six VDR SNPs (rs7975232, rs731236, rs2239185, and rs3782905) [14]. These results have demonstrated a replication of rs7975232 VDR-locus association with asthma among the Canadian women samples. However, the two polymorphisms rs731236 and rs3782905, which have reported a significant association in the NHS, have not shown any association with asthma in both Chinese population and Canadian families from the CAMP study. Elsewhere, Poon and colleagues addressed a study which involved a total of 223 independent French Northeastern Quebec families [13]. In contrast to our result, their results have not demonstrated any association between VDR rs7975232 association and asthma; however, six other VDR variants widely studied were significantly overtransmitted to both asthmatic and atopic offspring (p < 0.05) in the French families. Another study addresses the previously described association of VDR variants with asthma and related phenotypic traits, in which 951 German individuals from 224 pedigrees were included in the analysis [15]. Thirteen SNPs were tested and all reported no associations with asthma, including three non-significant SNPs also typed in our study (rs3782905, rs1544410 and the coding polymorphism rs731236).

Recently, researchers are following a key challenge to understand the functionality of different VDR polymorphisms, by the fact that most of these markers are synonymous. Most investigators have focused on the 3' regulatory region, because it is close to the synonymous markers tested mostly in association studies, such as BsmI, ApaI, and TaqI. The importance of N-terminal regions of the nuclear hormone receptors is increasingly recognized, especially through regulation of mRNA stability [25]. Only several papers have tried to identify the relation existing between significant associations and functional sequence variations in the VDR gene. Although no consistent association studies support those functionality findings in asthma disease.

## Conclusion

In summary, we identified a significant association between a genetic variant at the VDR locus and asthma in Chinese Han population. However, further studies are needed to find out the genuine functional variants in this region.

## Competing interests

The authors declare that they have no competing interests.

## Authors' contributions

QL conceived and designed the study. AS conducted the laboratory experiments, developed protocols, did the statistical analysis, and drafted the manuscript, GG helped genotyping, YG and QL polished the final version. CW and HL participated in the clinical survey and sample collections. All authors read and approved the final manuscript.

## Pre-publication history

The pre-publication history for this paper can be accessed here:


